# Impact of upgrading from a 25-cm to a 30-cm *z*-axis field of view digital PET/CT in a pediatric hospital

**DOI:** 10.1007/s00247-024-06049-6

**Published:** 2024-09-11

**Authors:** Joseph G. Meier, Andrew T. Trout, Nadeen Abu Ata, Susan E. Sharp, Christopher G. Anton, Elanchezhian Somasundaram, Samuel L. Brady

**Affiliations:** 1 Department of Radiology, Cincinnati Children’s Hospital Medical Center, 3333 Burnet Ave, MLC 5031, Cincinnati, OH 45229 USA; 2 Department of Radiology, University of Cincinnati College of Medicine, 3230 Eden Ave, Cincinnati, OH 45267 USA; 3 Department of Pediatrics, University of Cincinnati College of Medicine, 3230 Eden Ave, Cincinnati, OH 45267 USA; 4 Department of Radiology, AdventHealth Central Florida, Orlando, FL USA

**Keywords:** Decreased radiation dose, Decreased acquisition time, Increased PET sensitivity, Pediatric, Positron emission tomography

## Abstract

**Background:**

Increased positron emission tomography (PET) scanner *z*-axis coverage provides an opportunity in pediatrics to reduce dose, anesthesia, or repeat scans due to motion.

**Objective:**

Recently, our digital PET scanner was upgraded from a 25-cm to a 30-cm *z*-axis coverage. We compare the two systems through National Electrical Manufacturing Association (NEMA) testing and evaluation of paired images from patients scanned on both systems.

**Materials and methods:**

NEMA testing and a retrospective review of pediatric patients who underwent clinically indicated 18F-fluorodeoxyglucose (FDG) PET computed tomography (PET/CT) on both systems with unchanged acquisition parameters were performed. Image quality was assessed with liver signal to noise ratio (SNR-liver) and contrast to noise ratio (CNR) in the thigh muscle and liver with results compared with an unpaired *t*-test. Three readers independently reviewed paired (25 cm and 30 cm) images from the same patient, blinded to scanner configuration.

**Results:**

Expansion to 30 cm increased system sensitivity to 29.8% (23.4 cps/kBq to 30.4 cps/kBq). Seventeen patients (6 male/11 female, median age 12.5 (IQR 8.3–15.0) years, median weight 53.7 (IQR 34.2–68.7) kg) were included. SNR-liver and CNR increased by 35.1% (IQR 19.0–48.4%) and 43.1% (IQR 6.2–50.2%) (*P*-value <0.001), respectively. All readers preferred images from the 30-cm configuration. A median of 1 (IQR 1–1) for fewer bed positions was required with the 30-cm configuration allowing a median of 91 (IQR 47–136) s for shorter scans.

**Conclusion:**

Increasing *z*-axis coverage from 25 to 30 cm on a current-generation digital PET scanner significantly improved PET system performance and patient image quality, and reduced scan duration.

**Supplementary Information:**

The online version contains supplementary material available at 10.1007/s00247-024-06049-6.

## Introduction

One of the most significant advancements in positron emission tomography (PET) imaging has been the transition from analog detectors, such as conventional photomultiplier tubes, to digital detectors, like silicon photomultipliers [1]. This digitization, combined with enhancements in time-of-flight (ToF) resolution, has led to a substantial improvement in the signal-to-noise ratio (SNR) of PET images [1]. As a result, scan times can be shortened, and the amount of injected radioactive tracer reduced, making the process more efficient and patient-friendly. This paradigm shift from analog to digital PET detectors marks a pivotal moment in the evolution of PET imaging technology. More recently, manufacturers have been increasing the axial (craniocaudal) coverage of PET scanners [2–5]. Increased axial coverage improves scanner sensitivity, by capturing a higher percentage of the photons that are emitted from the patient, and allows for fewer bed positions to be acquired per patient, or for whole-body scanning in a single-bed position. For the pediatric population, these advances are of great potential importance. The higher scanner sensitivity potentially allows image acquisition with lower injected activity and therefore lower radiation exposure [6], and allows for improved image quality, while the greater axial field of view allows faster scanning time [7, 8]. Reductions in the total scan time reduce the incidence of motion artifacts which can render an exam non-diagnostic or require additional radiation exposure from repeat PET computed tomography (PET/CT) scans [9, 10]. Reductions in scan time also have the potential to reduce use or duration of sedation or general anesthesia which the youngest and non-compliant patients commonly require to avoid patient motion. Both sedation and anesthesia carry immediate risks due to the technical challenges of anesthesia administration as well as the potential interference with neurocognitive development in the youngest patients [11, 12]. The addition of anesthesia creates scheduling and workflow challenges, increased cost, and increased risk to the patient.

Recently, our hospital upgraded our existing digital PET/CT from an axial extent of five rings (25 cm) to the current maximum configurable length of six rings (30 cm) through the manufacturer’s addition of a ring to the existing detector assembly. A comparison of the technical performance between the 25- and 30-cm systems has previously been performed using National Electrical Manufacturing Association (NEMA) NU 2–2018 testing standards [13] and demonstrated improvements in sensitivity, image noise, and count rate performance. That study also showed a few patient cases to demonstrate the promise of the system to decrease imaging time and dose, or improve dynamic PET imaging using multi-bed position acquisitions. However, real-world evidence of the patient-care benefits of expanding the axial field of view of an installed PET scanner remains sparse. Our purposes were to (1) validate technical performance gains following expansion of an installed digital PET/CT from a 25-cm to a 30-cm axial field of view using key NEMA NU 2–2018 performance tests and (2) compare objective image quality metrics, subjective image quality assessments, and workflow improvements between patients scanned on the 25-cm and 30-cm scanner configurations.

## Methods

In this study, we compared performance of a Discover MI Generation 2 PET/CT, General Electric Healthcare, Chicago, IL, scanner configured with either five rings (25-cm axial coverage) or, after on-site upgrade, six rings (30-cm axial coverage). Both configurations used detectors with lutetium-based crystals coupled to Hamamatsu digital silicon photomultipliers (dSiPMs) [13]. Detectors were arranged in 34 detector modules with five or six detector units for the five- and six-ring systems respectively. Each detector unit was composed of a 16 × 9 array of crystals, with each crystal having dimensions of 3.95 mm (transaxial) × 5.3 mm (axial) and 25 mm (depth); in this configuration, the maximum allowed reconstructed transverse field of view is 70 cm.

### Physics testing

PET performance tests were performed according to the NEMA NU 2–2018 testing standard [14] using the vendor-provided acquisition protocols and vendor-provided acquisition processing tools. The tests relevant to and reported in this investigation were for sensitivity and image quality.

#### Sensitivity

The sensitivity test determined the number of coincidence events detected per second (cps) with respect to the radioactivity in kilobecquerel (kBq) present in the PET field of view. To acquire this data, a 70-cm plastic line source was filled with 3.66 MBq of fluorine-18 (F-18) calibrated for the beginning of the first acquisition. The dead time factor for the first acquired frame was 1.02, below the required value of 1.05 needed to minimize the loss of coincidence events. For each test, five 1-min acquisitions were performed: the first with the plastic tube inside of an aluminum tube, and for each subsequent acquisition, an additional concentric aluminum tube of increasing diameter was added. The cps/kBq for the five acquisitions were then extrapolated to give the value with no attenuation, which is defined as the scanner sensitivity. This test was performed at the isocenter and with a 10-cm offset vertically from the isocenter.

#### Image quality in phantom

To assess image quality, the NEMA International Electrotechnical Commission (IEC) body phantom was used. This phantom contained a 10,000-ml background, six fillable spheres of varying diameters (10 mm, 13 mm, 17 mm, 22 mm, 28 mm, and 37 mm), and a lung insert. The phantom was prepared with a 4:1 sphere to background ratio. At the starting time of PET data acquisition, the background activity was 5.33 kBq/ml. To simulate out of field scatter as would occur in a patient scan, the NEMA scatter phantom was placed adjacent to the body phantom, and the line source was filled with 116.8 MBq F-18 calibrated for scan time. A CT was acquired for attenuation correction (CTAC) and three consecutive PET acquisitions were performed with increasing durations of 383 s, 399 s, and 414 s to account for activity decay. PET reconstructions were performed with four iterations, 32 subsets, 2-mm Gaussian filtration, and ToF.

### Patient imaging

Patient images had been clinically acquired at an academic tertiary pediatric medical center. Patient images were retrospectively reviewed as part of this study. Our institutional review board provided an exempt determination for this study and waived the requirement for patient informed consent.

**Fig. 1:**
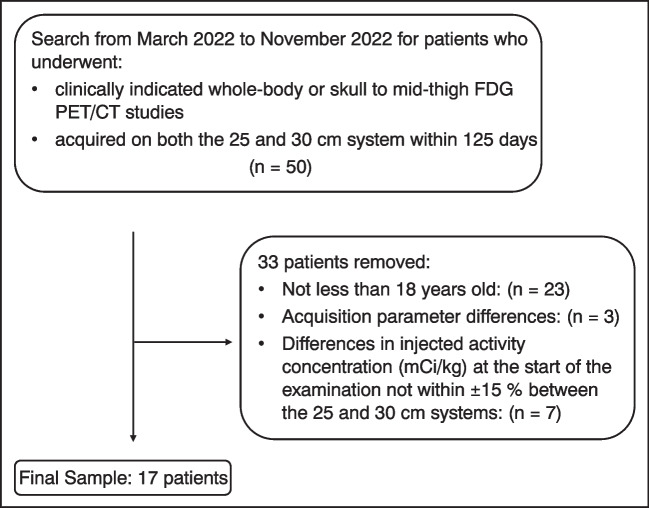
Flow diagram for patient selection. The *box* on the right corresponds to the patients that were removed

An imaging report search engine (Illuminate InSight) was used to identify patients who had undergone clinically indicated 18F-fluorodeoxyglucose (FDG) whole body or skull to mid-thigh PET/CT from March 2022 to November 2022, and who were scanned both before and after the system upgrade (*n*=50). During this period, the maximum interval between examinations for an individual patient was 125 days, which we considered acceptable to avoid bias due to changes in patient habitus. From the 50 identified patients, patients were then excluded for the following reasons: not less than 18 years old (*n*=23) and differences in acquisition parameters (*n*=3). In addition, to reduce bias-related differences in radiopharmaceutical activity, patients were excluded if the injected activity concentration (mCi/kg) at the start of both scans was not within ± 15% (*n*=7). This yielded 17 patients for the study (Fig. 1).

Patient data for both scans are listed in Table 1 and there were no significant differences. Per clinical protocol, the administered activities for 18F-FDG are 0.12 mCi/kg for patients > 20 kg and 0.10 mCi/kg for patients <20 kg with a minimum dose of 1 mCi and a maximum dose of 10 mCi during the study period. Patients were imaged from the vertex to feet after intravenous injection of 0.12 ± 0.01 mCi/kg 18F-FDG with uptake times of 57.8 ± 10.0 min. PET acquisition and reconstruction parameters were the same in both scanner configurations, except for the number of overlapping slices which was increased per the manufacturer’s recommendation from 23 (26%) to 31 (29%) for the 25-cm to 30-cm systems, respectively. Each PET bed position was acquired for 90 s, or 135 s, if MotionFree data-driven respiratory gating [15] automatically triggered respiratory motion correction. CTAC acquisitions were acquired with weight-based acquisition parameters using tube current modulation. PET reconstruction was performed with a Q.Clear Beta value of 700 [16] and using ToF, point spread function correction, 256 × 256 matrix, and a 70-cm FOV.

**Table 1:** Patient demographics

	Age (years)	Weight (kg)	Injected activity at scan time (mCi/kg)
5 rings	11.6 ± 4.0	50.0 ± 22.4	0.09 ± 0.01
6 rings	11.9 ± 3.9	52.4 ± 24.1	0.08 ± 0.01
*P*-value	0.85	0.71	0.31
Interval between scans (days)	90.7 ± 21.4		
Female	11		
Male	6		

Values are provided in mean ± standard deviation. No statistically significant differences were seen between scans

#### Objective image quality measurements in patients

To measure image quality in patient images across scanner configurations, the signal to noise ratio (SNR) was measured in the healthy liver while avoiding major vasculature. A board-certified medical physicist with 9 years of experience in PET imaging made all measurements under the guidance of a radiologist who was board certified in pediatric radiology and nuclear radiology with 11 years of post-fellowship experience. A 3-cm-diameter spherical region of interest was used for measurements of the mean and standard deviation of the standardized uptake value (SUV). SNR was calculated according to Eq. 1:

1 $$\text{SNR}=\frac{\text{mean}(\text{SUV liver})}{\sigma (\text{SUV liver})}$$

Contrast to noise ratio (CNR) measurements were made comparing liver signal to muscle signal based on measurements at the level of the mid-thigh using a 1-cm spherical region of interest. CNR was calculated according to Eq. 2:

2 $$\text{CNR}=\frac{\text{mean}\left(\text{SUV liver}\right)-\text{mean}(\text{SUV mid thigh})}{\sigma (\text{SUV liver})}$$

#### Subjective image quality assessments in patients

Three readers who regularly interpret pediatric PET/CT (16 years, 11 years, and 3 years of experience) blindly reviewed pairs of patient image series. Paired patient image series acquired on the 25-cm and 30-cm field of view scanners were displayed side-by-side, randomly positioned left and right, and reviewers chose their preferred image series. Two reading sessions were held. In the first session, the readers indicated their preference based on overall image quality. In the second session, the readers indicated their preference based first on liver homogeneity, and then on liver boundary delineation, and finally based on brain gray:white delineation. Two image scoring sessions were performed to reduce the likelihood that scoring overall image quality would bias the other image quality metrics. The order of display of individual patient image series and the left:right positioning of the 25-cm and 30-cm image series were re-randomized for the second session. Each image series pair was scored as follows: left preferred, left slightly preferred, no preference, right slightly preferred, and right preferred. These ratings were then decoded as 25-cm configuration preferred, 25-cm configuration slightly preferred, no preference, 30-cm configuration slightly preferred, and 30-cm configuration preferred. To assess intra-reader reliability, four patients were repeated after all patients were scored with the patient order and left:right positioning of the 25-cm and 30-cm image series re-randomized.

#### Workflow improvement assessment

To quantify the impact on patient workflow, for each patient, the difference in PET exam duration and number of beds required was measured.

### Statistical analysis

The normality of the CNR and SNR data was assessed with the Shapiro–Wilk test and an unpaired, two-tailed *t*-test was performed to assess the differences in SNR and CNR. To assess the differences in preference scores between scanner configurations, the Mann–Whitney *U* test was performed. To account for multiple hypothesis testing, Bonferroni corrections were applied to the objective image quality scores, as well as the subjective image quality scores. *P*-values less than 0.05 were considered statistically significant.

Fleiss’ kappa with quadratic weighting [17] was used to assess inter-reader agreement on preference based on each of the specific image quality assessments. To assess intra-reader reliability, a weighted Cohen’s kappa test was performed for each reader. The kappa values were interpreted as follows: <0 (poor agreement), 0.0–0.20 (slight agreement), 0.21–0.4 (fair agreement), 0.41–0.60 (moderate agreement), 0.61–0.80 (substantial agreement), 0.81–1.0 (almost perfect agreement) [18]. All statistical tests were performed in RStudio (2023.06.1), Posit Software, Boston, MA.

## Results

### Physics testing

**Fig. 2:**
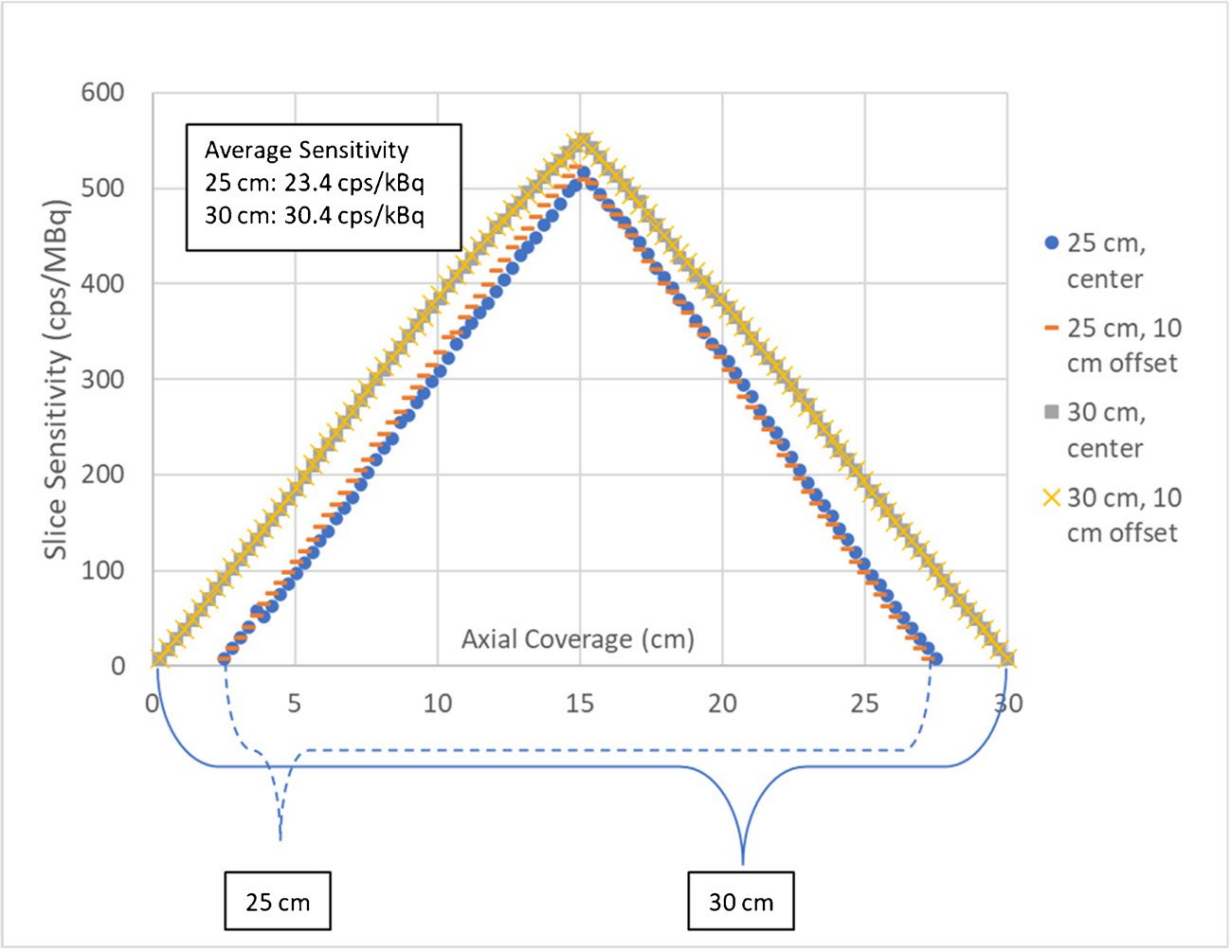
Plot of the sensitivity across the *z*-axis field of view of the 25-cm and 30-cm system configurations at the center (R 0) and at 10-cm (R 10) offset on the *y*-axis. The 5-cm increase results in a higher overall sensitivity

As seen in Table 2 and Fig. 2, the average sensitivity at the center and 10-cm offset was 29.8% greater for the 30-cm versus 25-cm scanner configuration. The 30-cm configuration additionally produces an increased contrast ratio for all spheres except for the 37-mm sphere, decreased background variability, and decreased lung error.

**Table 2:** National Electrical Manufacturing Association (NEMA) sensitivity and image quality test results

System	25 cm	30 cm	% change
Sensitivity			
At center (cps/kBq)	23.2	30.3	30.9%
10 cm (cps/kBq)	23.6	30.4	28.8%
Image quality contrast ratio			
10 mm	39.6 ± 2.8	47.4 ± 0.0	19.7%
13 mm	46.9 ± 2.0	53.0 ± 3.0	13.0%
17 mm	58.3 ± 2.3	63.9 ± 0.8	9.6%
22 mm	70.4 ± 1.5	70.4 ± 2.6	0.0%
28 mm	75.8 ± 1.0	79.4 ± 1.0	4.7%
37 mm	89.4 ± 1.2	79.1 ± 1.2	-11.5%
Image quality background variability			
10 mm	11.9 ± 0.6	7.4 ± 0.0	-37.8%
13 mm	9.8 ± 0.6	5.5 ± 0.5	-43.9%
17 mm	8.8 ± 0.5	4.0 ± 0.1	-54.5%
22 mm	7.6 ± 0.2	3.3 ± 0.2	-56.6%
28 mm	6.8 ± 0.1	2.7 ± 0.1	-60.3%
37 mm	6.3 ± 0.2	1.9 ± 0.0	-69.8%
Average lung error (std) (%)	11.5 ± 0.2	3.6 ± 0.08	-68.7%

#### Objective image quality measurements

**Fig. 3:**
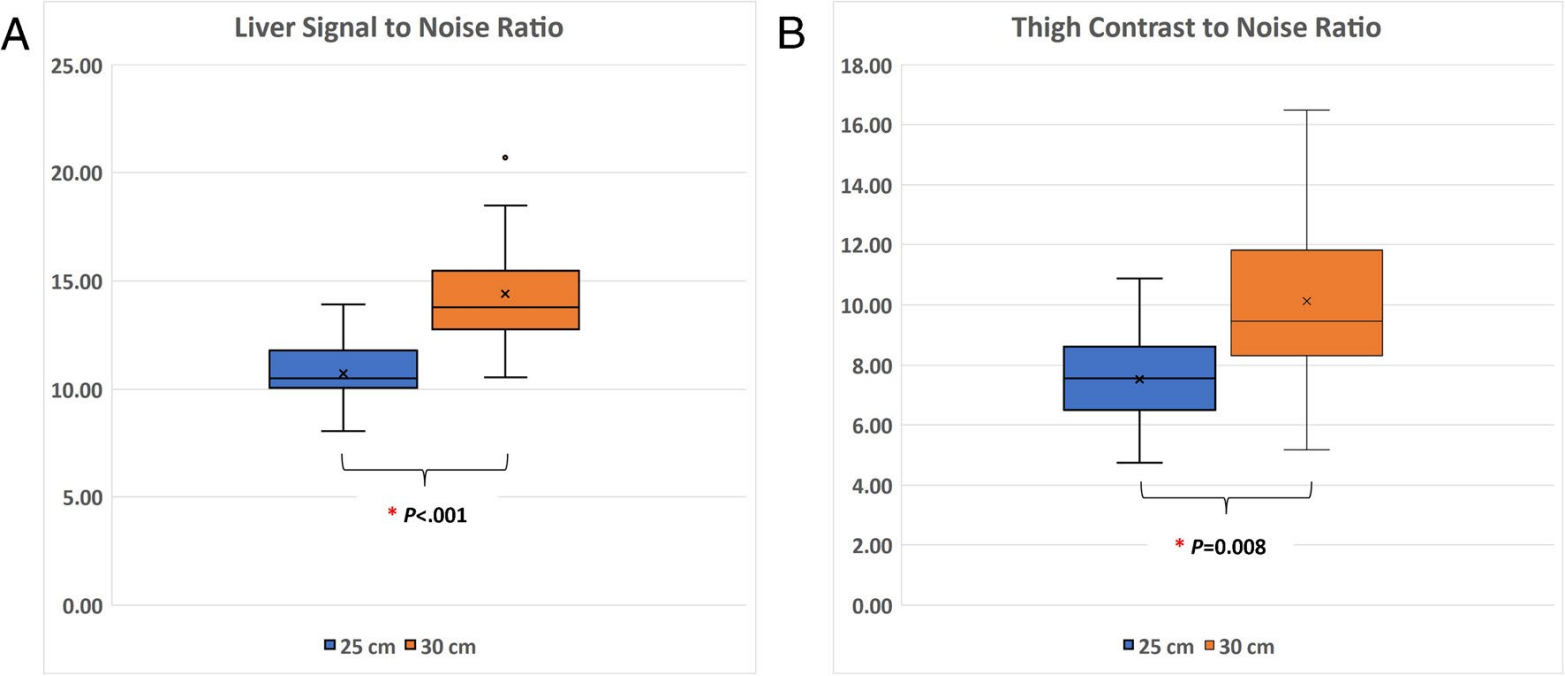
Measurements of (**A**) liver signal to noise ratio and (**B**) thigh muscle contrast to noise ratio with statistically significant improvements on the 30-cm scanner versus the 25-cm scanner

The SNR in the liver was 35.0% higher for the 30-cm scanner configuration (10.7 ± 1.5 vs. 14.4 ± 2.7; *P*-value <0.001), and CNR was 34.6% higher for the 30-cm scanner configuration (7.5 ± 1.6 vs. 10.1 ± 3.0; *P*-value =0.008), as demonstrated in Fig. 3.

#### Subjective image quality assessments

**Fig. 4:**
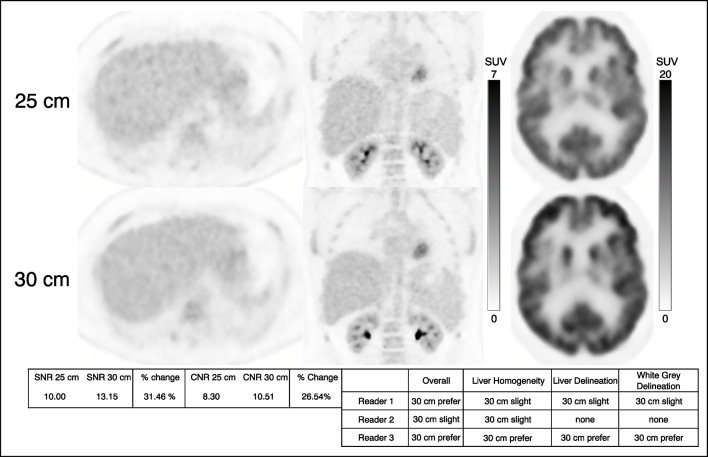
PET axial (*left*) image of the liver, coronal (*middle*) image of the abdomen and thorax, and axial view of the brain (*right*) from a 16-year-old girl. The patient’s weight, injected activity concentration, and uptake time were 54.3 kg, 0.12 mCi/kg, and 48.5 min on the 25-cm system and 56.7 kg, 0.12 mCi/kg, and 60 min for the 30-cm system. The ratio of the activity concentration at the start examination time of the 30-cm system to the 25-cm system was 0.92. Signal to noise ratio (SNR) and contrast to noise ratio (CNR) values improved with the 30-cm scanner configuration. All readers either slightly preferred or preferred images from the 30-cm scanner configuration except for two no preference ratings

**Fig. 5:**
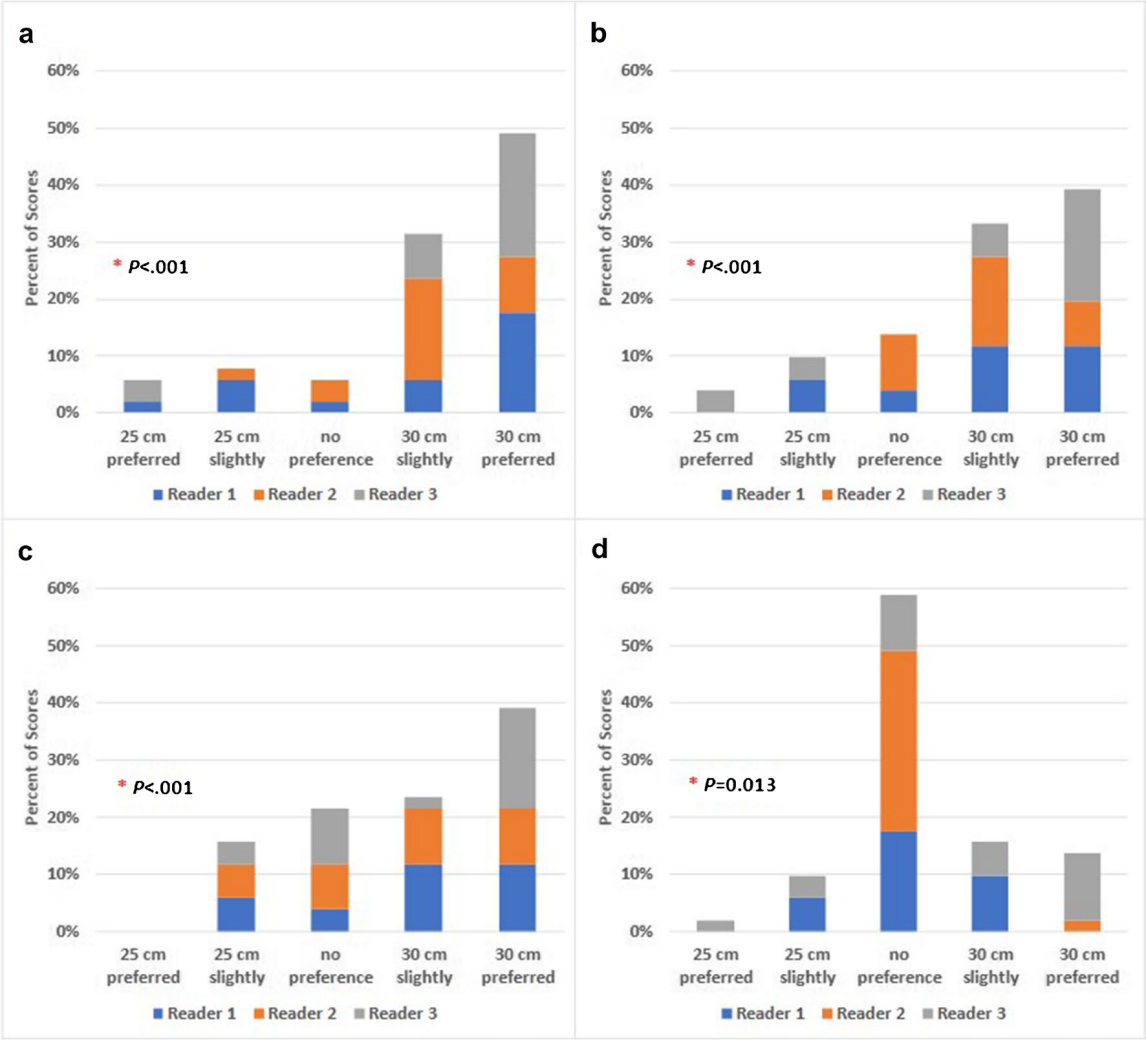
Observer preference for images from the 25-cm vs. 30-cm system configurations based on (**a**) overall image quality, (**b**) liver homogeneity, (**c**) liver boundary delineation, and (**d**) brain gray:white delineation. *P*-values <0.05 indicate a statistically significant difference in the distribution of scores between systems

When indicating preference based on overall image quality and liver homogeneity, all readers showed preference for images from the 30-cm scanner configuration (Fig. 4). Preference based on liver boundary delineation showed slightly more preference (63.0% preferred or slightly preferred) for images from the 30-cm scanner configuration. However, when indicating preference based on brain gray:white differentiation, most cases were scored as no preference. For all subjective image quality assessments, the differences in distributions of scores between scanner configurations were statistically significant (*P*-value <0.001) and for brain gray:white differentiation (*P*-value =0.013), as demonstrated in Fig. 5.

Inter-reader agreement on image quality metrics was moderate to substantial at 0.52 (0.09–0.85) for overall image quality, 0.47 (0.16–0.75) for liver homogeneity, 0.70 (0.53–0.85) for liver border delineation, and 0.57 (0.43–0.71) for gray:white differentiation. Measurements of intra-reader reproducibility with Cohen’s weighted kappa for all measurements resulted in scores of 0.38 (-0.07 to 0.83), 0.72 (0.52–0.92), and 0.40 (0.25–0.54) for readers 1, 2, and 3 respectively.

#### Workflow improvement assessment

**Fig. 6:**
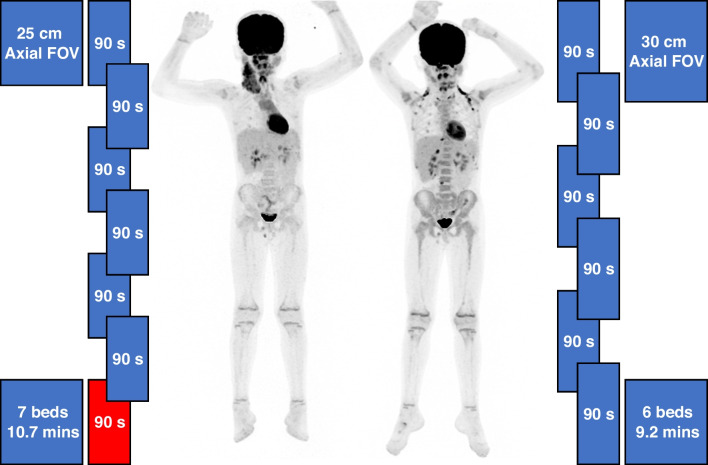
PET maximum intensity projection images of a 7-year-old boy scanned on the 25-cm system (*left*) and the 30-cm system (*right*). The patient’s weight, injected activity concentration, and uptake time, respectively, were 22.4 kg, 0.11 mCi/kg, and 62.3 min on the 25-cm system and 23.8 kg, 0.12 mCi/kg, and 68.7 min for the 30-cm system. The ratio of the activity concentration at the start examination time of the 30-cm system to the 25-cm system was 1.05. The images acquired with the 30-cm system configuration show decreased noise. The 5 cm of additional axial coverage required one less bed position, and therefore a decrease in scan time of 90 s. The window width and level are the same in both images (min 0, max 4 standardized uptake value)

On average, 1.06 ± 0.43 fewer beds were required when imaging the same patients on the 30-cm versus 25-cm system configurations and the overall scan duration decreased by 86.4 ± 63.6 s (-12.8%). A diagrammatic representation of these improvements is seen in Fig. 6.

## Discussion

This work demonstrates improvements in image quality gained by upgrade of a 25-cm axial field of view to a 30-cm axial field of view system PET scanner in a pediatric practice. Improvements are demonstrated in phantoms, and in terms of both objective and subjective image quality assessments in a group of patients imaged on both systems. We also show improvements in workflow through shorter scan durations and a need for fewer PET bed positions.

Conversion of the 25-cm scanner to a 30-cm configuration resulted in an increase in sensitivity of 29.8%, from 23.4 cps/kBq to 30.4 cps/kBq based on NEMA measurements. From this increase in acquired counts, it follows that the background variability (image noise) measured in the phantom decreased when scanning on the 30-cm system. These increases in sensitivity and decreases in image noise translated into improvements in image quality in pediatric patients where liver SNR increased by 35.0% and CNR in the leg increased by 34.6%. We did not assess the impact of the scanner upgrade on tumor CNR because the avidity and size of the tumors likely changed between scans on the two systems due to the effect of disease therapy. However, due to the measured improvements in image quality, we can infer that the detectability of tumors should be improved on the 30-cm system. In addition, should the user keep PET bed position durations the same, the lower noise in the image will introduce less bias into the measurement of SUVmax, the most frequently used semi-quantitative measurement of tumor activity.

Subjective image quality assessments favored the 30-cm system for all metrics; however, for brain gray:white differentiation, the difference in preference between the two systems was less substantial. Example pairs of patient images (Fig. 4) demonstrate that the liver is more homogenous and that the boundaries of the liver among other organs are better defined in images obtained on the 30-cm system, accounting for observer preferences. We posit that the less pronounced preference between systems for brain gray:white differentiation reflects the typical large difference in uptake between the metabolically active cortex and relatively less metabolically active white matter. It is possible that another metric such as noise in the cephalad and caudad regions of the brain might show less noise in the images obtained on the 30-cm system due to its longer field of view.

The potential for a reduction in patient injected dose or acquisition time is demonstrated for one patient imaged during the study period (Supplementary Material 1). This patient was excluded from the objective and subjective image quality assessments due to uptake time differences (46.9 min vs. 89 min) caused by logistical challenges related to prepping, sedating, and scanning a young patient that resulted in dissimilar activity concentrations in the patient at the time of scan. Consequentially, the 30-cm configuration scan had 39% less activity concentration (mCi/kg) at the beginning of the scan in comparison to the 25-cm configuration scan. Objective image quality assessment demonstrated SNR and CNR differences of only 1.9% to 2.5%, respectively, and subjective observer assessment determined only slight to no preference for the 30-cm configuration images. This suggests that for this patient, injected patient dose or scan time could be reduced by 38.5% when scanning on the 30-cm scanner.

The final metrics considered are the improvements in workflow with respect to the number of bed positions, total scan time, and potential for patient dose reduction. Due to the increased geometrical coverage, on average, one less bed position was required in our patient sample resulting in an average 90-s decrease in acquisition time. On a per-patient basis, a longer imaged field of view has particular implications. For smaller pediatric patients, a decrease of one bed position from three beds to two beds means a 33.3% decrease in scan time which might change the way a patient is managed with respect to anesthesia administration. For the tallest patients of 1.6 m and 1.7 m in height, the increased field of view allows acquisition of two fewer bed positions which reduced the scan time by 3 min. For these taller patients who are older and are more capable of holding still, the primary benefit to the patient is comfort based on faster scan throughput.

Our results suggest that an upgrade of an existing PET scanner from 25 to 30 cm provides a 29.8% increase in sensitivity which could be leveraged to reduce dose to the patient or reduce scan time by the same percentage with no loss in image quality. In our practice, we injected on average 0.12 mCi/kg, which could be reduced to 0.084 mCi/kg when leveraging the increased sensitivity. This is below the currently recommended injected activity range of 0.1–0.14 mCi/kg specified by the 2021 SNMMI Procedure Standard/EANM Practice Guideline on Pediatric 18F-FDG PET/CT for oncology and suggests the need to revisit these recommendations given the trend in increasing scanner sensitivity [19]. Opportunities to reduce scan time open the possibility to scan certain patients awake who would have needed anesthesia to remain still. This reduces the risks of administration of anesthesia to the patient and in addition makes these procedures less complicated and less expensive by not needing additional staffing and equipment in the examination room. The shorter scan time will likely benefit patients who are suffering from high levels of anxiety which could be related to developmental issues, claustrophobia, or concerns related to their diagnosis and treatment. Alternatively, for institutions with a large number of scans performed per day, reduction in scan times should allow more patients to be scanned in 1 day. Finally, increased sensitivity could also be leveraged to acquire images with less noise which would improve lesion conspicuity, or for smaller patients to increase the reconstruction matrix size without as high an increase in noise.

A prior investigation has shown that through optimization of reconstruction parameters, PET bed durations could be reduced from 90 to 60 s on the 25-cm field of view scanner without significantly impacting image quality [20]. Applying the same reconstructions to the 30-cm field of view scanner should theoretically allow a reduction in the acquisition time per bed position to 42 s. For the smallest patients requiring two bed positions, this would allow a PET acquisition of less than 90 s. These potential reductions in scan time are certainly not as substantial in comparison to those afforded by the long axial field of view PET systems which can now cover 106 cm to 194 cm [2, 3]. However, from a financial perspective, for the majority of radiology departments, a relatively shorter axial field of view scanner, such as the scanner under investigation in this paper, would most likely be considered.

Our study is limited by the separation in time (average 90 days) between acquisitions on the 25-cm and 30-cm systems. Patients might have changed in habitus, disease state, or treatment regimen during this period impacting image quality assessment. Also, we routinely employ a respiratory motion correction algorithm, MotionFree [15], which automatically detects and corrects for respiratory motion. Some patients might have had differences in whether respiratory motion correction was applied between the two scans which may have impacted scan duration and image quality. Additionally, the impact on lesion quantification and conspicuity could not be assessed because the disease state would be different between scans. To assess the intra-reader reproducibility, only four exams were repeated, which reduces the validity of the result. Finally, this study did not investigate the impact on brain PET or any other radiotracers; however, it is expected that the increase in sensitivity will have the same positive impact.

## Conclusion

In conclusion, an increase in *z*-axis field of view from 25 to 30 cm on a General Electric DMI system results in a significant improvement in system performance, image quality, and scan duration. Future work will determine how we will best leverage the increase in sensitivity to reduce injected activity, reduce scan time, or acquire images with improved image quality which will benefit our pediatric patient population.

## Supplementary Information

Below is the link to the electronic supplementary material.

Supplementary file1 (JPG 1284 KB)

## Data Availability

The datasets analyzed during the study are available from the corresponding author on reasonable request.
