# Supplementary figures and images for: Impact of upgrading from a 25-cm to a 30-cm z-axis field of view digital PET/CT in a pediatric hospital
Source: Pediatr Radiol. 2024 Sep 11;54(11):1896–905. doi: 10.1007/s00247-024-06049-6 (PMC11473537; doi:10.1007/s00247-024-06049-6)

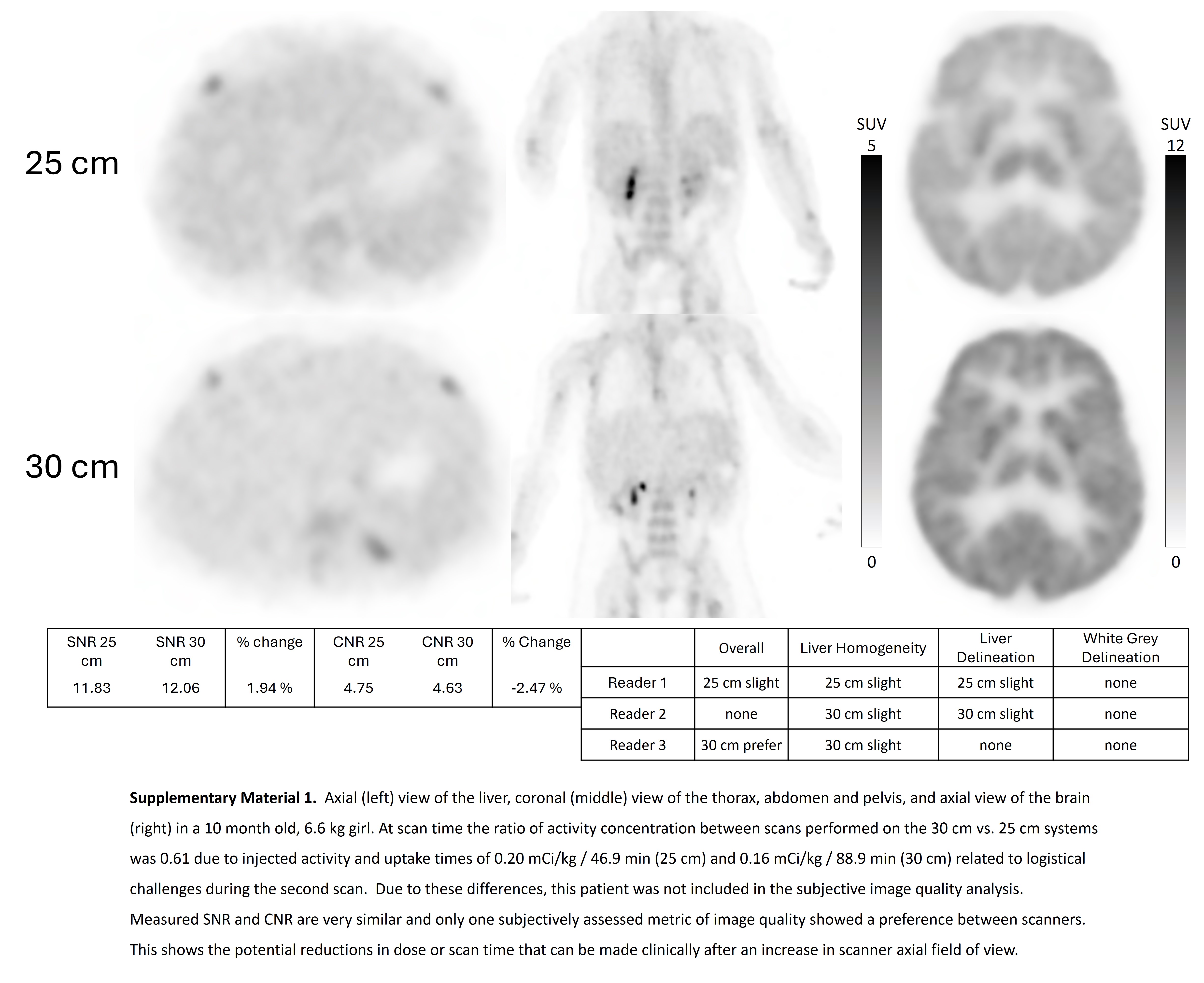

Supplement: Supplementary file 1 — Supplementary file1 (JPG 1284 KB) [file 247_2024_6049_MOESM1_ESM.jpg]
